# Prophylactic and therapeutic neutralizing monoclonal antibody treatment prevents lethal yellow fever infection

**DOI:** 10.1172/jci.insight.191665

**Published:** 2025-07-15

**Authors:** Lauren N. Rust, Michael J. Ricciardi, Savannah S. Lutz, Sofiya Yusova, Johan J. Louw, Aaron Yrizarry-Medina, Sreya Biswas, Miranda Fischer, Aaron Barber-Axthelm, Gavin Zilverberg, Lauren Bailey, Tonya Swanson, Rachael Tonelli, G.W. McElfresh, Brandon C. Rosen, Thomas B. Voigt, Christakis Panayiotou, Jack T. Mauter, Noor Ghosh, Jenna Meanor, Giovana Godoy, Michael Axthelm, Jeremy Smedley, Mark K. Slifka, Esper G. Kallas, Gabriela Webb, Robert Zweig, Caralyn S. Labriola, Benjamin N. Bimber, Jonah B. Sacha, David I. Watkins, Benjamin J. Burwitz

**Affiliations:** 1 Oregon National Primate Research Center (ONPRC), Oregon Health and Science University, Beaverton, Oregon, USA.; 2 Mabloc LLC, Washington DC, USA.; 3 George Washington University, Washington DC, USA.; 4 Vaccine and Gene Therapy Institute, Oregon Health and Science University, Beaverton, Oregon, USA.; 5 Department of Infectious Diseases and Tropical Medicine, School of Medicine, University of São Paulo, São Paulo, Brazil.; 6 Butantan Institute, São Paulo, Brazil.

**Keywords:** Immunology, Infectious disease, Hepatitis, Therapeutics

## Abstract

Yellow fever virus (YFV) infection is fatal in 5%–10% of the 200,000 yearly cases. There is currently no available antiviral treatment. We showed previously that administration of 50 mg/kg of a YFV-specific neutralizing monoclonal antibody (nmAb) at 2 days postinfection (dpi), prior to the onset of severe disease, protected YFV-infected rhesus macaques from death. To further explore the clinical applicability of our nmAb MBL-YFV-01, we treated rhesus macaques with a lower dose (10 mg/kg) of this nmAb prophylactically or therapeutically at 3.5 dpi. We show that a single prophylactic or therapeutic i.v. dose of our nmAb protects rhesus macaques from death following challenge. A comprehensive analysis of 167 inflammatory cytokine and chemokines revealed that protection was associated with significantly reduced expression of 125 of these markers, including type I IFN, IL-6, and CCL2. This study further expands the potential clinical use of our YFV-specific nmAb, which could be used during an outbreak for immediate prophylactic immunity or for patients with measurable serum viremia.

## Abstract 2

Yellow fever virus is fatal in 5–10% of infections. We show that administration of a neutralizing monoclonal antibody can prevent fatal infection.

## Introduction

Yellow fever virus (YFV) continues to affect those living in areas with large mosquito populations and limited vector control, resulting in endemicity in 47 countries (1). Urbanization of previously uninhabited areas of South America, along with climate change–driven expansion of mosquito habitats, have put an increasing number of unprotected people at risk for infection (2, 3). There are 200,000 YFV cases reported annually (1). However, due to the underreporting of cases, the WHO estimates actual case numbers to be 10–250 times higher than currently reported (1, 4). Approximately 15% of YFV-infected individuals will progress to severe disease, and among this group, 30%–60% will die (4).

During an outbreak of YFV, vaccination campaigns are essential. There is a clinically available live-attenuated vaccine against YFV (YFV-17D and derivatives) (5); however, there are several contraindications (e.g., pregnancy, age, immune status). Furthermore, vaccination can cause 2 extremely rare but sometimes fatal complications known as yellow fever vaccine–associated viscerotropic disease and yellow fever vaccine–associated neurotropic disease, resulting in vaccine hesitancy (6–9). Moreover, approximately 20% of vaccinated individuals do not have neutralizing antibodies by 10 years after vaccination, in conflict with the WHO recommendation that protection is life-long (10, 11). It has been shown that detectable levels of anti-YFV antibodies provided by the YFV vaccine do not arise until at least 10 days after vaccination (10). This lag in protection leaves populations of people vulnerable to infection during an active outbreak.

Severe yellow fever generally follows a biphasic disease progression. In the acute phase of infection, symptoms include fever, headache, jaundice, and muscle and joint pain. Symptoms generally resolve within 4 days, and 85% of patients clear infection. For the remaining patients, progression into the intoxication phase can result in hemorrhage, multiorgan failure, coma, and death (12, 13). Given the biphasic nature of yellow fever progression, there are clear opportunities in a clinical setting to deploy therapeutic drugs to ameliorate severe disease.

Unfortunately, there is currently no clinically available antiviral treatment for YFV-infected individuals; patients are given simple symptom management and palliative care. Neutralizing monoclonal antibody (nmAb) treatment is a promising antiviral option due to its specificity, increasing ease of production, and high efficacy of viral neutralization (14). nmAb therapy can also be used both prophylactically and therapeutically (15).

There was a single human phase I clinical trial of an anti–YFV IgG nmAb treatment reported in 2020 (16), but no phase II trial has been announced to date. A major limitation of this phase I clinical trial was that the nmAb was only tested against YFV-17D and not against a pathogenic strain of the virus. We have previously shown that administering a high-dose of YFV-specific nmAbs to YFV-infected rhesus macaques (RMs) 2 days postinfection (dpi) resulted in no detectable disease and 100% survival (17). Importantly, nmAb treatment will provide immediate protection against infection, which can be critical during YFV outbreaks. Here, we expand on these promising results by exploring YFV-specific nmAb administration both prior to YFV challenge and therapeutically at time points with measurable serum viremia.

## Results

### Study design.

**Figure 1:**
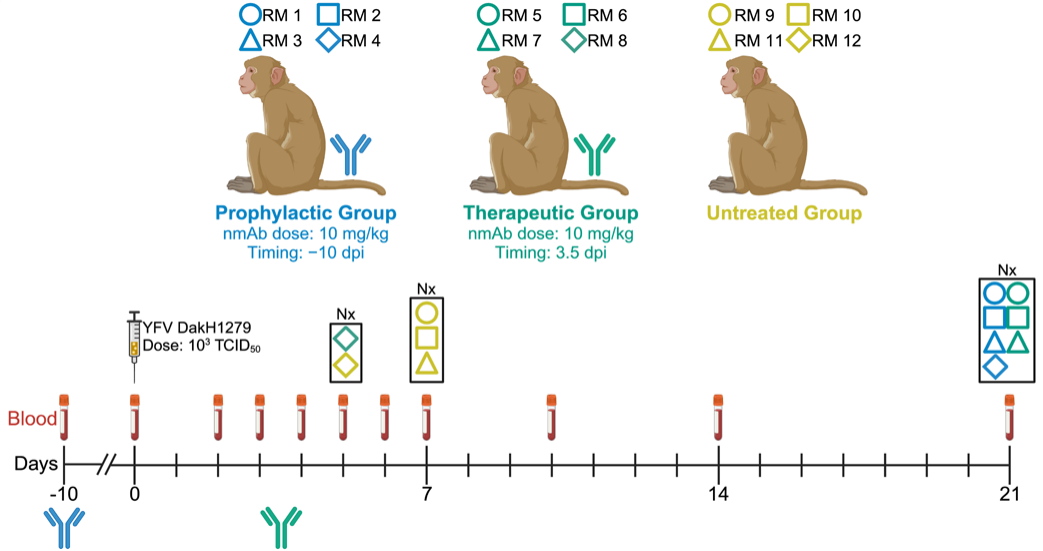
Study design for the testing of MBL-YFV-01 in YFV-DakH1279-infected RMs.

Our previous work demonstrated the efficacy of YFV-specific nmAbs in preventing severe disease and death in YFV-infected RMs when administered i.v. 2 dpi at a dose of 50 mg/kg (17). We sought to further expand the clinical applicability of one of these nmAbs, MBL-YFV-01, by testing it prophylactically and at a postinfection time point with detectable serum viremia. We selected 3.5 dpi as our therapeutic time point based on historical data from untreated, YFV-infected RMs, which first had detectable serum viremia on either 3 or 4 dpi. We assigned 12 RMs to 3 experimental groups based on their nmAb treatment: prophylactic (RM 1, RM 2, RM 3, and RM 4), therapeutic (RM 5, RM 6, RM 7, and RM 8), and untreated (RM 9, RM 10, RM 11, and RM 12) (Figure 1 and Supplemental Table 1; supplemental material available online with this article; https://doi.org/10.1172/jci.insight.191665DS1). We challenged all RMs with 1,000 TCID_50_ of the highly pathogenic macaque-adapted strain YFV-DakH1279 (18). All 12 RMs were challenged with the same dose of YFV-DakH1279 regardless of weight, sex, or age. It has been previously demonstrated that the logarithmic replication that occurs with yellow fever in this model is not affected by the challenge dose (19), indicating that weight is not a confounding variable. Comparing serum YFV loads from historical untreated RMs and untreated RMs from this study and categorizing by weight shows no significant difference of weight on viremia (Supplemental Figure 1A) (17). There has yet to be an in-depth analysis on the effect of sex on survival from YFV in RMs, but our historical data from all YFV-challenge studies indicate no significant difference when comparing serum YFV loads and categorizing by sex (Supplemental Figure 1B) (17). Clinically, age has been shown to affect survival from YFV, with increased age increasing the risk for mortality from YFV (20). Comparing serum YFV loads from historical untreated RMs and RMs from this study and categorizing by age suggest that age does not affect viremia (Supplemental Figure 1C) (17). The prophylactic treatment group received 10 mg/kg MBL-YFV-01 i.v. at –10 dpi, while the therapeutic treatment group received 10 mg/kg MBL-YFV-01 i.v. at 3.5 dpi to mimic treatment in the intoxication phase.

### Prophylactic and therapeutic nmAb treatments prevent severe yellow fever disease.

**Figure 2:**
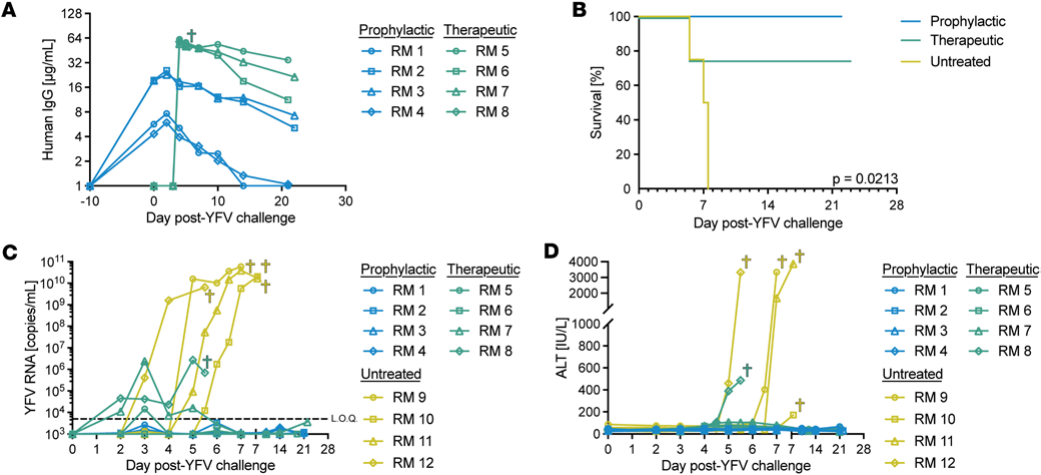
Prophylactic and therapeutic administration of MBL-YFV-01 protects RMs from lethal YFV infection. (**A**) Longitudinal concentration of MBL-YFV-01 in the plasma of YFV-DakH1279 challenged RMs. (**B**) Kaplan-Meier survival curves of RMs after challenge with YFV-DakH1279 and treatment with YFV-specific antibodies. *P* value determined by Mantel-Cox test with Bonferroni correction. (**C**) Longitudinal serum YFV-DakH1279 loads in RMs. LOQ, 5 × 10^3^ copies/mL. (**D**) Longitudinal serum ALT levels in RMs.

We measured the concentration of MBL-YFV-01 in the plasma of all 8 RMs in both treatment groups. Prophylactically treated RMs had concentrations of nmAb between 4.3 and 19.5 μg/mL at the time of YFV challenge, while all animals in the therapeutic group achieved concentrations ranging from 54.5 to 61.7 μg/mL (Figure 2A). MBL-YFV-01 has an in vitro IC_50_ of 12.2 ng/mL against YFV DakH1279 (17). We achieved in vivo levels ranging from 352- to 5,057-fold above its in vitro IC_50_. All prophylactically treated RMs and 3 of 4 therapeutically treated RMs survived through the study endpoint of 21 dpi, while all 4 untreated animals had to be euthanized due to severe disease by 7 dpi (*P* = 0.0213) (Figure 2B). We found that all 4 untreated RMs had high serum viral loads (sVL) of > 1 × 10^9^ RNA copies/mL (range: 1.87 × 10^9^ to 1.18 × 10^11^) at the time of euthanasia (Figure 2C). In contrast, none of the RMs in the prophylactic group had detectable sVL above the limit of quantification (LOQ) following YFV challenge (LOQ: 5 × 10^3^ YFV RNA copies/mL). Three of 4 RMs in the therapeutic group had detectable serum viremia at 3 dpi. RM 7 peaked at 3 dpi with a sVL of 2.41 × 10^6^ RNA copies/mL that fell below the LOQ by 6 dpi. sVL in RM 8 peaked at 5 dpi with 2.71 × 10^6^ RNA copies/mL that declined to 6.99 × 10^5^ RNA copies/mL by the time of euthanasia at 5.5 dpi due to clinical endpoints.

Alanine transaminase (ALT) levels indicate liver pathology and are used clinically to monitor hepatic infections. All 4 RMs in the prophylactic group had ALT levels within the normal RM range of 18.9–94.2 IU/L throughout the study (Figure 2D). Three RMs in the therapeutic group showed slightly elevated ALT levels that remained below 110 IU/L (Supplemental Figure 2), but RM 8 reached a clinical endpoint ALT of 486 IU/L at 5 dpi. Three untreated RMs had elevated ALT values (>3,000 IU/L) that were consistent with acute hepatic necrosis associated with severe viscerotropic yellow fever infection. RM 10 had a rising ALT of 172 IU/L, an sVL of 1.99 × 10^10^ RNA copies/mL, and other pathophysiological characteristics of disease (see below) on 7 dpi going into an evening with severe inclement weather. Therefore, the ethical decision was made to euthanize this animal prior to reaching a clinical endpoint.

### Prophylactic and therapeutic nmAb treatments reduce YFV replication in the tissues.

**Figure 3:**
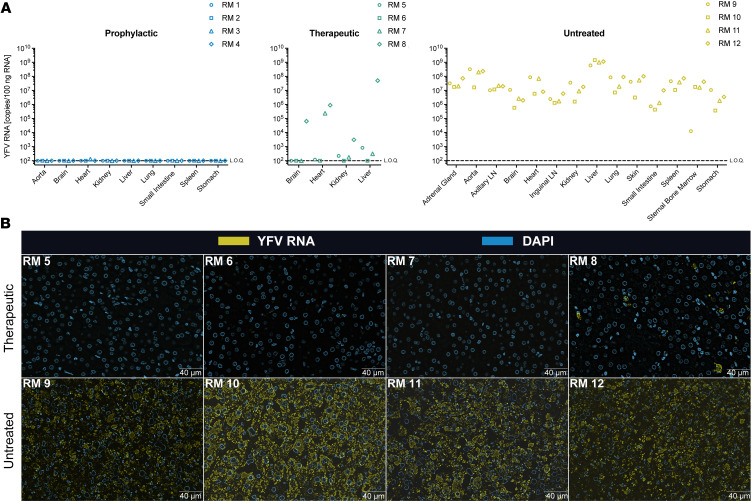
YFV RNA expression in the tissues. (**A**) Quantification of YFV-DakH1279 RNA by RT-PCR in necropsy tissues. LOQ, 1 × 10^2^ copies/100 ng RNA. Day of necropsy for each animal: RM 1–21 dpi, RM 2-21 dpi, RM 3–21 dpi, RM 4–21 dpi, RM 5–19 dpi, RM 6–19 dpi, RM 7–22 dpi, RM 8–5.5 dpi, RM 9–7 dpi, RM 10–7.5 dpi, RM 11–7.5 dpi, and RM 12–5.5 dpi. (**B**) RNAscope staining of YFV-DakH1279 RNA in the livers of YFV-DakH1279-infected RMs. Scale bars: 40 μm.

We extracted RNA from multiple tissue types at necropsy to define the anatomical distribution of YFV. All 4 RMs in the prophylactic group had tissue YFV RNA below the LOQ (Figure 3A). In contrast, we found YFV RNA in the brain (1 of 4), hearts (3 of 4), kidneys (3 of 4), and livers (3 of 4) of RMs in the therapeutic group. We also detected YFV RNA in all tissue types from the 4 untreated RMs (Figure 3A). The highest levels of YFV RNA were detected in the livers of these RMs (6.00 × 10^8^ to 1.44 × 10^9^ copies/100 ng RNA), with high levels of YFV RNA also detected in the adrenal glands, aortas, axillary lymph nodes, brains, hearts, inguinal lymph nodes, kidneys, lungs, skin, small intestines, spleens, femoral bone marrow, and stomachs. Importantly, these RMs were euthanized at different time points after YFV challenge. Therefore, direct comparisons of tissue YFV RNA need to be performed with caution. These data suggest that YFV may be replicating outside the liver in RMs, although given the high sVL, this may also be detection of YFV RNA in the blood perfusing the tissues.

To address this question in more detail, we determined which organs supported YFV replication. We used RNAscope to locate YFV RNA in the livers of therapeutic and untreated RMs. YFV RNA was found in nearly all hepatocytes of the 4 untreated RMs, correlating with the high number of YFV RNA copies found in liver tissues by quantitative PCR (qPCR) (Figure 3B). In contrast, only RM 8 from the therapeutic group had detectable YFN RNA in the liver, matching the positive sVL present at the time of euthanasia (Figure 3B). Additionally, we tested for YFV RNA in the cerebellums, hearts, lungs, spleens, and kidneys of all 4 untreated RMs. We found YFV RNA in the cerebellums (3 of 4), hearts (2 of 4), lungs (4 of 4), spleens (4 of 4), and kidneys (4 of 4) of untreated RMs (Supplemental Figure 3). In contrast, we did not detect YFV RNA in any of these tissues from a naive RM.

### Pathophysiological measures indicate severe disease in untreated YFV-infected RMs.

**Figure 4:**
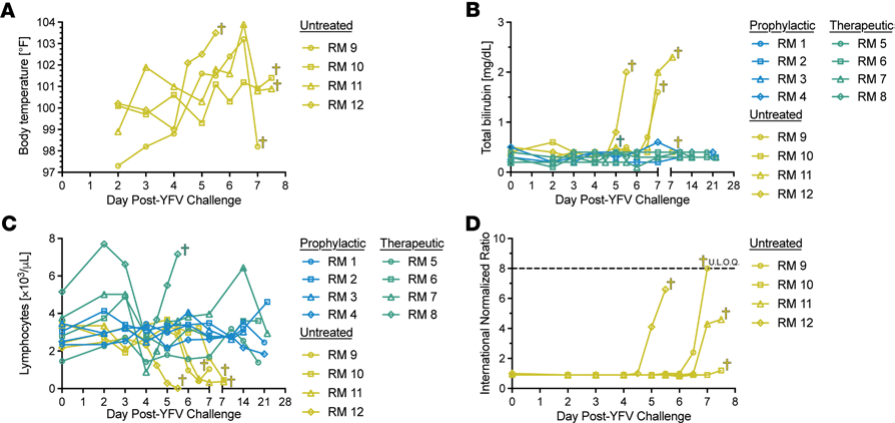
Pathophysiology of YFV-DakH1279 infection in RMs. (**A**) Body temperatures. (**B**) Total bilirubin levels. (**C**) Blood lymphocyte counts. (**D**) International normalized ratio measurements.

We next monitored the clinical and pathophysiological markers of YFV infection in untreated RMs (Supplemental Table 2). All 4 untreated RMs experienced fevers, with temperatures peaking shortly before or at the time of euthanasia (Figure 4A). We found that 3 of 4 untreated RMs exhibited bilirubin levels exceeding 1.5 mg/dL (normal range 0.3–0.5 mg/dL) on 6 and 7 dpi, indicative of excess RBC breakdown, hepatobiliary injury, and general liver dysfunction (Figure 4B) (21). Lymphopenia is a hallmark of YFV infection that precedes hepatic enzymopathy; we found severe lymphopenia in all 4 untreated RMs within 48 hours of euthanasia (Figure 4C) (14). Microscopically, germinal centers displayed lymphoid apoptosis, necrosis, and increased tingible body macrophages in multiple lymphoid organs such as the spleen, tonsils, lymph nodes, and gut-associated lymphoid tissue from 3 of 4 untreated RMs (Supplemental Figure 4 and Supplemental Table 3). In the therapeutic group, RM 7 experienced transient lymphopenia (0.88 10^3^/μL) at 4 dpi, 0.5 days after treatment, which resolved within 8 hours and returned near baseline by 5 dpi. Though values remained within reference ranges, RM 5, RM 6, and RM 8 had a lymphocytic nadir at 4–5 dpi (1.42 × 10^3^/μL to 2.62 × 10^3^/μL). In contrast, RMs in the prophylactic group exhibited no lymphopenia.

We also looked for evidence of coagulopathy in the untreated RMs, as there is a dearth of information on the underlying mechanisms of coagulopathy in humans with severe yellow fever. We measured the International Normalized Ratio (INR), a standardization for prothrombin time, across the study time points in our untreated animals. INR defines the rate of blood clot formation, with high values indicating a clotting deficiency. All 4 untreated RMs had normal INR values (0.90–1.12) at the time of YFV challenge, but INR values increased rapidly with disease progression, and the INR spike in RM 9 exceeded the detector limit of 8 (Figure 4D). To supplement intensive monitoring for coagulopathy, a clinical scoring rubric was used. Reflecting hepatic disease, clinical symptoms were not apparent until ALT exceeded 1,000 IU/L, which was present in 3 of 4 animals (score of 7–12) (Supplemental Table 4). The most common clinical signs were lethargy, hyporexia/nausea, and pallor.

Histopathology of the liver correlated with hematologic parameters, with no significant findings in the prophylactic and 3 therapeutically treated RMs. Minimal midzonal necrosis with Councilman bodies (areas of hepatocyte degeneration, hallmark of YF infection), were present in RM 8, and similar lesions of mild severity were seen in RM 10. The remaining untreated animals had massive hepatic necrosis and hemorrhage, splenic congestion and neutrophilic inflammation in the marginal zone and red pulp, and renal tubular degeneration with protein with or without cellular casts (Supplemental Figures 4–6, and Supplemental Table 3). Additionally, RM 9 and RM 12 had gall bladder edema and pancreatic acinar vacuolation with loss of zymogen granules (Supplemental Figure 4). Altogether, these data indicate that the pathophysiological features of severe yellow fever in RMs mirrors those in patients and that our model may be able to define the mechanisms underlying disease progression.

### Inflammatory cytokines and chemokines are associated with severe yellow fever infection.

There are very few reports of cytokine/chemokine analyses in the plasma or serum of patients with severe yellow fever (22, 23). Advances in technology now allow for the simultaneous measurement of hundreds of cytokines/chemokines from a single sample (24). We therefore set out to define the cytokine/chemokine profile associated with severe yellow fever in our RM model. We performed nucleic acid linked immuno-sandwich assay (NULISA) (24) on baseline (prior to YFV infection) and longitudinal post-YFV plasma samples from RMs, allowing us to define 167 inflammatory cytokines/chemokines down to attomolar concentration.

**Figure 5:**
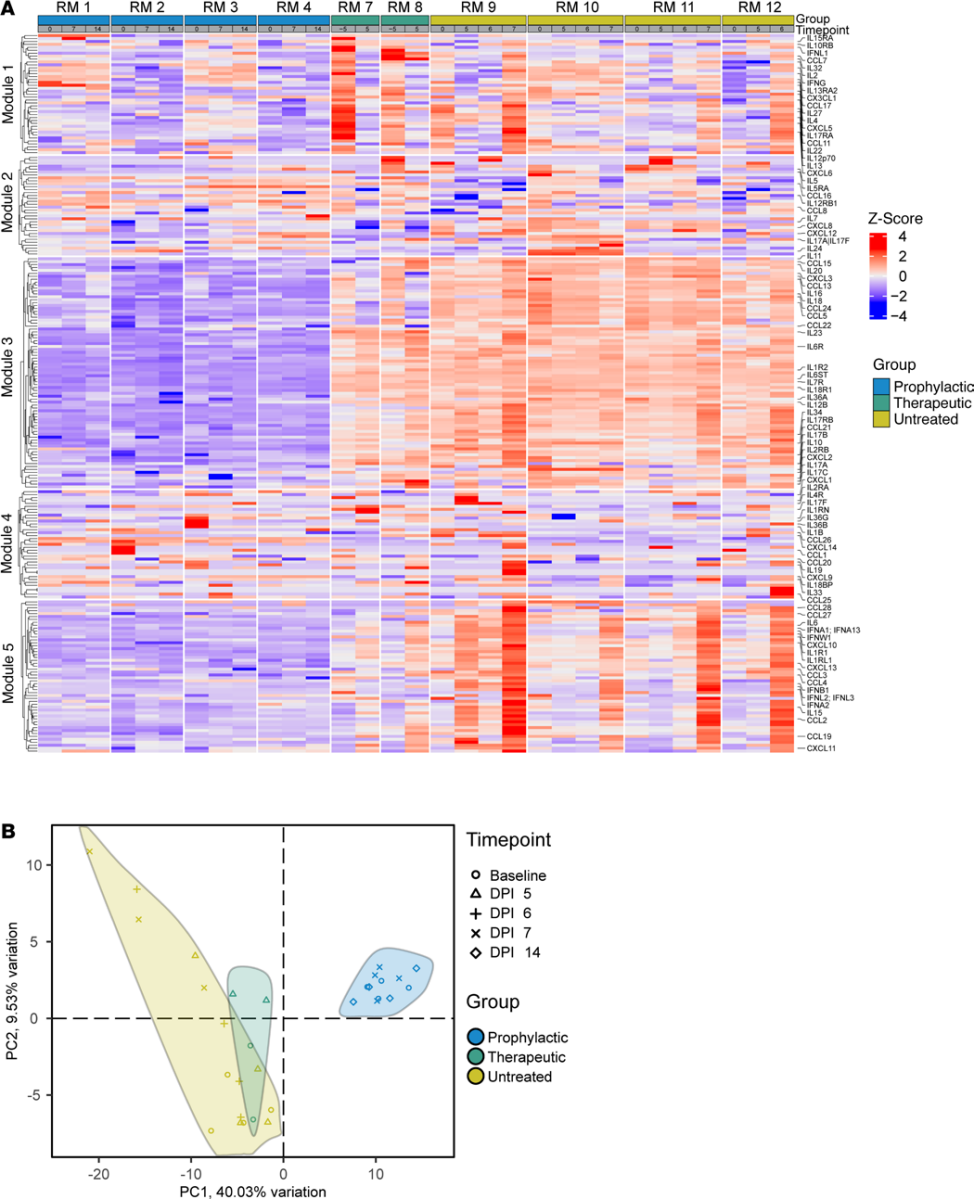
Inflammatory cytokine/chemokine profiles across treated and untreated RM groups. (**A**) Heatmap showing levels of inflammatory cytokines/chemokines in plasma across treatment groups. Rows are clustered via k means. (**B**) Principal component analysis showing the similarities between the inflammatory cytokine/chemokine profiles for each treatment group.

We found that each study group exhibited a unique profile, with untreated RMs generally having the highest abundance of inflammatory markers (particularly modules 3 and 5), the therapeutic group having a more modest abundance of similar markers, and the prophylactic group having a below average abundance of these markers (Figure 5A). Hierarchical clustering revealed several modules with distinct expression profiles. Module 1 corresponds to type II proinflammatory cytokines and chemokines. The untreated group showed a slight upregulation of these proteins over time. Module 2 contains many cytokines and chemokines that are induced by IL-1B, which is associated with broad, acute inflammation. Module 3 contains proteins within various IL families. The prophylactic group maintained a low abundance of these proteins, while the therapeutic and untreated groups showed a similar, widespread upregulation of these proteins over time. Module 4 corresponds with ILs that are involved with acute inflammation, specifically Th17 responses. Module 5 is composed of proteins belonging to or associated with type I IFN and where we saw the most drastic changes in expression over time. All 4 untreated RMs showed a large upregulation of proteins within this module. In contrast, the prophylactic group maintained a below-average abundance of these proteins, likely due to the nmAb treatment. The therapeutic group showed an upregulation of these proteins but attenuated compared with the untreated group, suggesting nmAb treatment dampens the inflammatory response seen in untreated YFV infection.

Principal component analysis of these data revealed similarities between the cytokine/chemokine profiles of untreated and therapeutic groups, while the prophylactic group was distinct (Figure 5B). This difference in the prophylactic group’s cytokine/chemokine profiles was evident even at the baseline time point, indicating a potential effect of bolus nmAb treatment at 10 days prior to YFV challenge. These data indicate that YFV infection induced similar inflammatory cytokine/chemokine responses across RMs and that our therapeutic administration of nmAb blunted this response.

**Figure 6:**
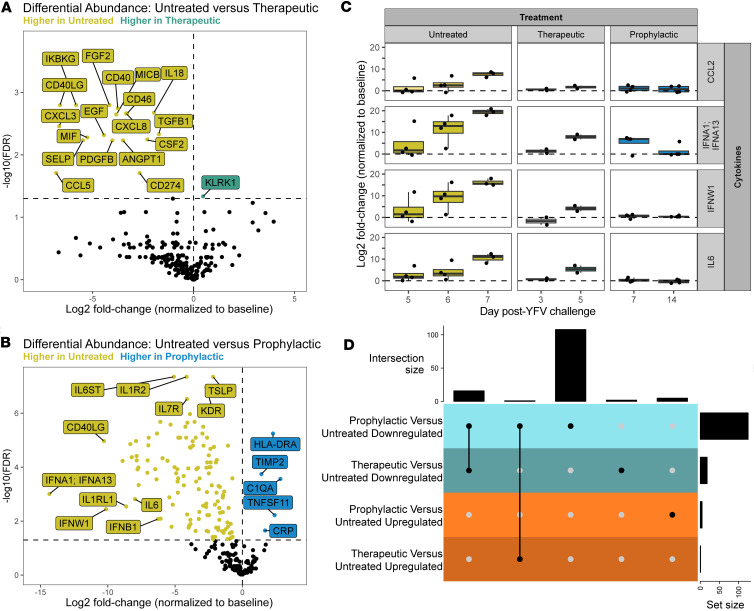
Plasma proteomics/NULISA differential abundance. (**A**) Volcano plot showing baseline-subtracted protein levels upregulated in the therapeutic group in green and upregulated in the untreated group in yellow as an average treatment effect over all time points. (**B**) Volcano plot showing baseline-subtracted protein levels upregulated in the prophylactic group in blue and upregulated in the untreated group in yellow as an average treatment effect over all time points. (**C**) Longitudinal expression of CCL2, IFNA1/IFNA13, IFNW1, and IL-6. (**D**) UpSet plot showing the number of shared (connected black dots) and unique (individual black dots) differentially abundant plasma proteins from the comparisons in **A** and **B**.

In-depth proteomics analysis of the NULISA data revealed differences in cytokine/chemokine abundance across groups compared with baseline (Figure 6, A and B). The only protein significantly upregulated in the therapeutic group versus the untreated group when compared with baseline was the NK cell activator killer cell lectin like receptor K1 (KLRK1) (Figure 6A). In contrast, several proteins were shown to be significantly upregulated in the prophylactic group compared with the untreated group: complement C1q A chain (C1QA), C-reactive protein (CRP), HLA-DRA, the metallopeptidase inhibitor TIMP2, and TNFSF11 (Figure 6B). Many proteins were significantly upregulated in the untreated group compared with both the prophylactic and therapeutic groups, including CCL2, IFNA1/13, IFNW1, and IL-6 (Figure 6C). The expression of CCL2 increased over time in the untreated and therapeutic groups, indicative of acute liver injury (25), while remaining the same in the prophylactic group. IFNA1/13 increased over time in both the untreated and therapeutic groups and was also elevated at 7 dpi in the prophylactic group but decreased to near baseline levels by 14 dpi in 3 of 4 RMs. IFNW1 and IL-6 increased in both the untreated and therapeutic groups, also indicative of acute liver injury (26), while no changes in these cytokines were observed in the prophylactic group. The UpSet plot of these data revealed that the therapeutic and prophylactic groups, when compared with the untreated group, shared many of the same downregulated proteins (Figure 6D). In contrast, the prophylactic group had large numbers of uniquely downregulated cytokines/chemokines in comparison with the untreated group that were not shared when comparing the therapeutic and untreated groups.

**Figure 7:**
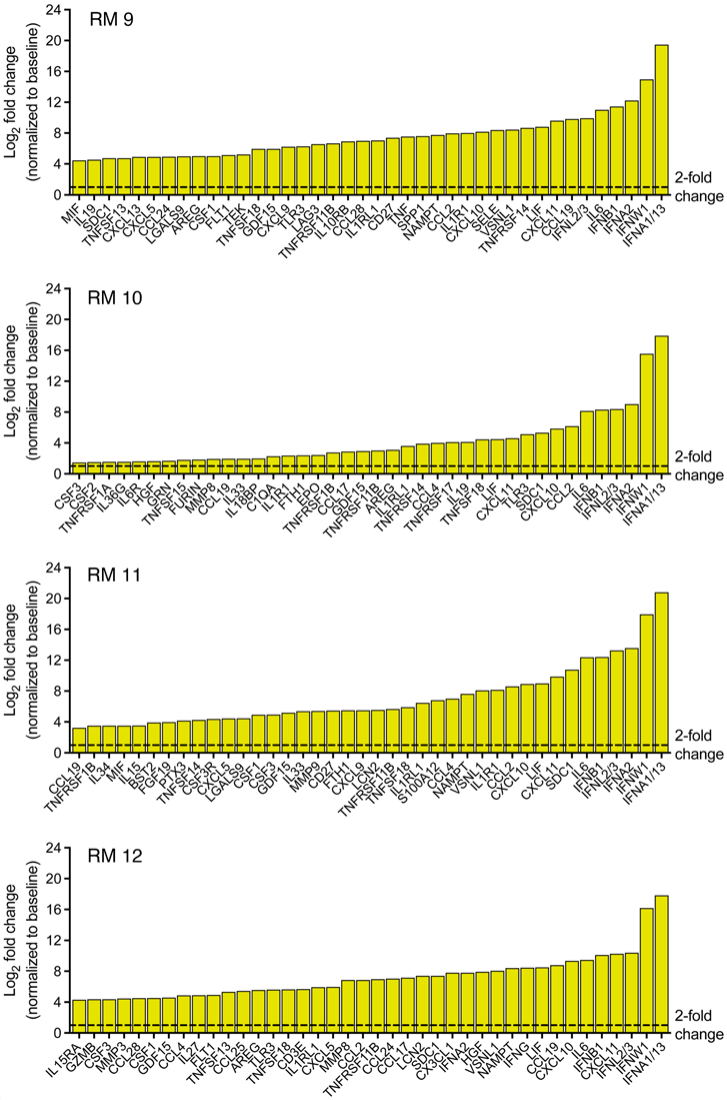
Top 40 expressed inflammatory cytokines/chemokines in untreated, YFV-DakH1279-infected RMs.

We were next interested in defining the largest inflammatory cytokine/chemokine responses in untreated RMs during severe yellow fever infection. We identified the 40 cytokines/chemokines with the greatest upregulation between baseline and euthanasia for each untreated RM (Figure 7). The top 2 upregulated cytokines in all untreated animals were IFNA1/13 (231,461- to 1,844,747-fold change) and IFNW1 (32,150- to 251,771-fold change), both critical innate immune signaling cytokines with significant antiviral properties. Next, we compared the top 10 upregulated cytokines/chemokines and found strong upregulation of IFNL2/3, IL-6, and IFNB1 in all animals with severe yellow fever. These results showcase the significant inflammatory cytokine/chemokine profile observed in RMs with severe yellow fever and further indicate that our treatment diminishes these inflammatory responses.

## Discussion

With only palliative care available for severe yellow fever and increasing levels of vaccine hesitancy, there is a clear need for an effective antiviral treatment. These data expand on our previous success of preventing severe yellow fever with therapeutic administration at 2 dpi of 50 mg/kg MBL-YFV-01 (17) by showing that a reduced dose of 10 mg/kg is effective as both a prophylactic or therapeutic treatment. Furthermore, we show that this lower dose given at 3.5 dpi reduces viremia and incidence of death. This broadens the applicability of MBL-YFV-01 while also reducing the cost of providing it clinically.

All 4 prophylactically treated and 3 of 4 therapeutically treated RMs survived through ~3 weeks after infection. RM 8 was the only animal that received MBL-YFV-01 that required euthanasia because it reached a clinical end point. However, unlike all other RMs we have infected in this and previous studies, sVL were dropping at the time of euthanasia, and RNAscope of the liver at necropsy revealed lower levels of viral RNA in comparison with untreated RM livers. Therefore, although we cannot conclude whether this RM would have survived, we did observe antiviral effects indicative of the treatment.

YFV infections result in degeneration in multiple visceral organs, but it remains unclear if viral replication occurs in these tissues (27). We detected YFV RNA in all tissues collected from untreated RMs. However, given the high levels in the blood, it is probable that this RNA was from circulating virus in the blood. We therefore conducted a more detailed study of YFV RNA expression by in situ staining and found viral replication in the livers, brain, hearts, lungs, spleens, and kidneys of untreated RMs. Flaviviruses enter hepatocytes via clathrin-mediated endocytosis, but the entry receptor is still unknown (28). Because clathrin-mediated endocytosis is not exclusive to hepatocytes, multiple cell types may have the ability to support YFV replication, but this has yet to be reported in the literature (29). Our data indicate that YFV replication can occur outside of the liver during severe yellow fever infection and potentially contributes to the clinical sequelae, which include bradycardia, cardiovascular instability, and renal insufficiency.

We also tested for pathophysiological changes in YFV-infected RMs to monitor for signs of liver dysfunction, coagulopathy, and other blood disorders. All 4 untreated RMs exhibited classical signs of severe YFV infection, with fever, lymphopenia, and high levels of ALT and bilirubin. These values correlated with the pathology in the livers at necropsy, marked by pallor and necrosis, degree of germinal center lymphoid necrosis, and renal tubular degeneration. Neutrophilic splenitis and degeneration of the pancreatic acini, present in 3 of 4 and 2 of 4 of the untreated RMs, respectively, have not been previously noted in macaques. Neutrophilic splenitis has additionally not been described in humans or animal models of YFV. Neutrophil activation and infiltration play a role in disease caused by other flaviviruses, such as Japanese encephalitis, though the mechanisms in YFV have yet to be fully explored (30). Pancreatic acinar degeneration has been noted in hamster models — though, to our knowledge, this study represents the first report in RMs (31). Severe YFV can cause pancreatitis in humans, with increased lipase being a prognostic indicator of disease progression (32). In a study by Bailey et al., no increases in lipase were seen in YFV-infected RMs (27). All 4 untreated RMs had high INR values, a hallmark sign of coagulopathy seen in patients with severe yellow fever. RM 8 was the only treated animal to exhibit symptoms of YFV infection, although the severity was diminished in comparison with untreated RMs.

In addition, we expanded upon previous studies of inflammatory cytokine/chemokine responses in YFV infection to show that there is an acute inflammatory cytokine storm in severe yellow fever infection that is mediated by IFNs, IL-6, CXCL10, CXCL11, and LIF (22, 23, 33). Importantly, these markers were also found in YFV-infected patients in Brazil using a Luminex 27-plex panel, supporting the relevance of our RM model (22). Indeed, our data support the previously published patient data while also providing a much more thorough analysis of 167 cytokine/chemokine responses in YFV infection.

We noted that there was a stark difference between the cytokine/chemokine profiles of the prophylactic and therapeutic groups. The inflammatory response seen in the therapeutic group is congruent with the clinical presentation of the RMs and their detectable sVL. Although administering nmAb therapeutically reduced sVL and prevented mortality in 3 of 4 RMs, the upregulation of inflammatory cytokines/chemokines indicate that our nmAb does not completely reduce the inflammatory response associated with YFV disease. In a clinical setting, it may be imperative to minimize inflammation while simultaneously neutralizing virus to prevent liver and other organ damage.

Concentrations of MBL-YFV-01 as low as 4.3 μg/mL at the time of YFV challenge were protective, demonstrating the potency of this nmAb. The dual application of nmAbs as both prophylactic and therapeutic treatments facilitates strategic flexibility in outbreak management, filling critical gaps left by traditional vaccines, especially when vaccine-induced immunity is suboptimal or when rapid immunity is required. While antibody-dependent enhancement (ADE) has been observed in people vaccinated with the pan-dengue vaccine Dengvaxia (34), YFV has only 1 serotype; therefore, ADE is not anticipated in the context of nmAb administration to previously 17D vaccinated individuals. However, there is some evidence to suggest that previous 17D vaccination results in ADE when the patient is infected with a different flavivirus (35). Further exploration into the effect our nmAb treatment has on subsequent flavivirus exposures will be needed to ensure cross-reactivity does not occur. The demonstrated potency of MBL-YFV-01 against all tested strains of YFV (including primary isolates) and these newer data at lower doses supports the commercial development of this nmAb (17). Inclusion of half-life extending nmAb mutations (e.g., YTE and/or LS mutations) and scalable production strategies will further enhance its viability as a cost-effective solution for broad clinical use in tandem with vaccines. Supporting investment and development in mAb technologies like MBL-YFV-01 could provide substantial public health benefits, curtail outbreaks, and offer essential treatment avenues for unvaccinated individuals or those for whom vaccines are contraindicated.

## Methods

### Sex as a biological variable.

Our study utilized both male and female RMs of Indian origin (Supplemental Table 1). However, due to unbalanced grouping, we are not powered to consider sex as a biological variable.

### YFV challenges and passive antibody administration.

RMs were challenged s.c. with 1 × 10^3^ TCID_50_ YFV-DakH1279 and assigned to 3 experimental groups: (a) i.v. treatment with 10 mg/kg antibody MBL-YFV-01 at –10 dpi (prophylactic group, *n* = 4), (b) 10 mg/kg MBL-YFV-01 at 3.5 dpi (therapeutic group, *n* = 4), or (c) untreated (untreated group, *n* = 4).

### Clinical and pathologic assessment.

Humane endpoint for treated animals was set by ALT > 300 IU/L and/or clinical condition at the discretion of attending veterinarians, and euthanasia was carried out for all animals in accord with the 2022 Edition of the American Veterinary Medical Association Guidelines for the Euthanasia of Animals. Due to advanced monitoring for coagulopathy and expected rapid progression of disease in the untreated group, a clinical rubric was established to augment hematologic assays, which included broad parameters of (a) overall clinical appearance particularly in reference to jaundice and hemorrhage, (b) respiratory and perfusion indicators, (c) activity and attitude, and (d) temperature during anesthesia (Supplemental Tables 2 and 4). Animals with a clinical score above 5, ALT over 150 IU/L, or sVLs exceeding 1 × 10^6^ YFV RNA copies/mL were evaluated every 2–4 hours. Preparation for imminent endpoint by end-of-day was initiated at ALT > 500 IU/L and immediate endpoints were set for clinical score of 10 and/or ALT > 1,000 IU/L. Tissues were collected at necropsy, with samples prepared for histopathologic analysis by fixation in 4% paraformaldehyde for 24 hours followed by 70% ethanol at 4°C for 4–6 days, paraffin embedding, sectioning at 5 μm, staining with H&E on a Leica ST5020 Autostainer, and scanned on a Leica AT2 slide scanner at ×20 or ×40 magnification. Slides were evaluated by 2 board-certified veterinary pathologists using Leica DM 3000 LED microscopes and HALO Link software (Indica labs).

### Quantification of delivered human IgG.

Human nmAb concentration was determined in RM plasma using the Human Therapeutic IgG1 ELISA Kit (Cayman Chemical, 500910) according to the manufacturer’s instructions. Briefly, heat-inactivated plasma samples and standards (provided with the kit) were diluted in assay buffer and added to 96-well α-human IgG1–precoated plates. Plates were covered and incubated for 2 hours at room temperature. Wells were washed 4 times with kit-provided wash buffer, before being fixed with 4% PFA for 15 minutes at room temperature. After fixation, wells were washed 4 times with kit-provided wash buffer. Therapeutic IgG Assay-HRP Conjugate (provided with the kit) was added to wells, and plates were incubated for 1 hour at room temperature. After incubation, wells were washed 4 times with kit-provided wash buffer. Kit-provided TMB Substrate was added to wells, and plates were incubated for 10 minutes at room temperature in the dark. After incubation, kit-provided Stop Solution was added to the plates. Plates were read on the Synergy HTX Multi-Mode Microplate Reader (BioTek), and data were collected using software Gen5 v3.09 at 2 absorbance wavelengths, 650 nm and 450 nm. The final OD was determined by subtracting OD_650 nm_ from OD_450 nm_. Final concentrations of human IgG1 in mg/mL were determined using a 4-parameter logistic curve fit.

### YFV-DakH1279 RNA quantification in serum.

Serum viral RNA was determined as previously described (17). YFV NS1 RNA from serum was quantified using the TaqPath 1-Step qPCR Master Mix (Thermo Fisher Scientific, A15299) using primers: YFV_qPCR-Forward (5′-GCAGGATCCAAAGAATGTTTACC-3′), YFV_qPCR-Reverse (5′-CCCAAGTCTTCCAACCATACT-3′), and YFV_qPCR-Probe (5′-6FAM-TTTCCAGAATTCGGGATGGTCTGC-TAMRA-3′) using an annealing temperature of 60°C. All manufacturer-defined thermocycling parameters were followed. All thermocycling and quantification analyses were conducted on an QuantStudio 3 (Applied Biosystems, A28567). Quantification was assessed relative to an absolute standard curve using synthesized RNA corresponding to the qPCR target region.

### YFV-DakH1279 RNA quantification in tissues.

Total intracellular DNA and RNA were extracted from tissues as previously described (17), YFV RNA from tissues was quantified using the TaqPath 1-Step RT-qPCR Master Mix (Thermo Fisher Scientific, A15299) using primers: YFV_qPCR-Forward (5′-CACGGGTGTGACAGACTGAAGA-3′), YFV_qPCR-Reverse (5′-CCAGGCCGAACCTGTCAT-3′), and YFV_qPCR-Probe (5′-6FAM-ATGGCGGTG/ZEN/AGTGGAGACGATTG-TAMRA-3′) using an annealing temperature of 60°C. All manufacturer-defined thermocycling parameters followed. All thermocycling and quantification analyses were conducted on an QuantStudio 3 (Applied Biosystems, A28567). Quantification was assessed relative to an absolute standard curve using synthesized RNA corresponding to the qPCR target region.

### Blood assays.

ALT, total bilirubin, and lymphocyte counts were determined as previously described (17). INR/Prothrombin Time (PT) was determined using the CoaguChek XS System (Roche Diagnostics).

### YFV RNA in situ hybridization.

RNA detection in tissues was performed using RNAscope as we have previously described (17).

### NULISA immunoassay.

Plasma samples were inactivated with 1% Triton-X (Sigma-Aldrich, X100) as described previously (36), before being sent to Alamar Biosciences for the NULISA assay. NULISA sampling and analysis were completed as described previously (24, 37). Briefly, plasma samples were added to a reaction mixture containing capture antibody cocktails and incubated at room temperature for 1 hour to allow immunocomplex formation. After incubation, 10× dT beads were added to the reaction mixture and incubated at room temperature for 1 hour to allow capture of the immunocomplex on the beads. After incubation, the bead immunocomplexes were collected by KingFisher Presto magnetic head (Thermo Fisher Scientific) and then washed. Beads were then removed, and streptavidin beads were added to eluent to recapture the immunocomplex. These beads were incubated with a ligation reagent LMM and a ligator sequence containing a unique sample barcode per sample to generate the ligated reporter oligonucleotide. The final barcoded immunocomplex was pooled into a library and amplified via 16 PCR cycles. The library was cleaned utilizing Ampure XP Reagent (Beckman Coulter) and quantified via Qubit. The library quantified by NGS on a NextSeq 1000/2000 instrument (Illumina) utilizing a P2 reagent kit for 100 cycles. Differential abundance analysis of the plasma proteomics/NULISA data was performed by fitting hierarchical generalized linear mixed models using maximum likelihood estimation and including a random intercept for each subject via lme4 (38). The data were baseline subtracted (0 dpi for the untreated and prophylactic groups, –5 dpi for the therapeutic group) prior to modeling, and all postinfection time points were pooled to eliminate mediation effects of disease progression (days after infection) on the average treatment effect of the antibody treatment on cytokine abundance.

### Statistics.

*P* value for survival curve (Figure 2B) was determined by Mantel-Cox test with Bonferroni correction. Statistical analysis for plasma proteomics and NULISA differential abundance (Figure 6) was completed using the following: Significance of the average treatment effect was computed by a likelihood ratio test, where the constrained model contained only a fixed intercept and random intercept per subject, and *P* values were adjusted using the Benjamini-Hochberg method (39). Statistical analysis was performed in R v4.4 and figures were generated using ggplot2 and ComplexHeatmap (40–42).

### Study approval.

Animals were cared for at the ONPRC with the approval of the Oregon Health and Science University’s IACUC using the standards of the *Guide for the Care and Use of Laboratory Animals* (National Academies Press, 2011). Euthanasia was carried out for all animals in accordance with the 2022 edition of the *American Veterinary Medical Association Guidelines for the Euthanasia of Animals*.

### Data availability.

All data are included in the manuscript, with raw data available from corresponding author upon request. Monoclonal antibodies are patented and available only with acceptable Material Transfer Agreement. Values for all data points in graphs are reported in the Supporting Data Values file.

## Author contributions

The co–first author order of LNR and MJR was based on the quantity and complexity of experiments conducted. This study was conceptualized by LNR, MJR, EGK, DIW, JBS, and BJB, all of whom contributed to the design of the experiments. Methods for assays utilized in this study were designed and written by LNR, MJR, GWM, GW, DIW, JBS, and BJB. Experiments were performed by LNR, SSL, SY, JJL, AYM, SB, MF, ABA, CP, JTM, NG, JM, GG, MA, JS, and RZ. RN sampling and care was provided by MF, ABA, GZ, LB, TS, RT, MA, JS, RZ, and CSL. Data validation was performed by LNR, MJR, BCR, TBV, BNB, and BJB, all of whom reviewed the primary data. Formal analysis of the primary data was conducted by LNR, MJR, GWM, and BJB. Critical resources for the RM experiments, including assay materials, antibodies, YFV-Dakar challenge stocks, and historical data were provided by MKS. The original draft of this manuscript was written by LNR and BJB. The manuscript was further reviewed and edited by MJR, BCR, MKS, CSL, DIW, BNB, JBS, and BJB. Graphs, pictures, tables, and all other visualizations were prepared by LNR, SB, GWM, and BJB. The study was supervised by principal investigators DIW, JBS, and BJB. Administration of the project was overseen by BJB. Funding for this study was acquired by DIW and JBS.

## Supplementary Material

Supplemental data

Supporting data values

## Version 1 07/15/2025

In-Press Preview

## Version 2 08/22/2025

Electronic publication
