# Supplementary material for: Prophylactic and therapeutic neutralizing monoclonal antibody treatment prevents lethal yellow fever infection
Source: JCI Insight. 2025 Jul 15;10(16):e191665. doi: 10.1172/jci.insight.191665 (PMC12416903; doi:10.1172/jci.insight.191665)
Supplement: Supplemental data [file jciinsight-10-191665-s008.pdf]

Supplemental Figure 1.

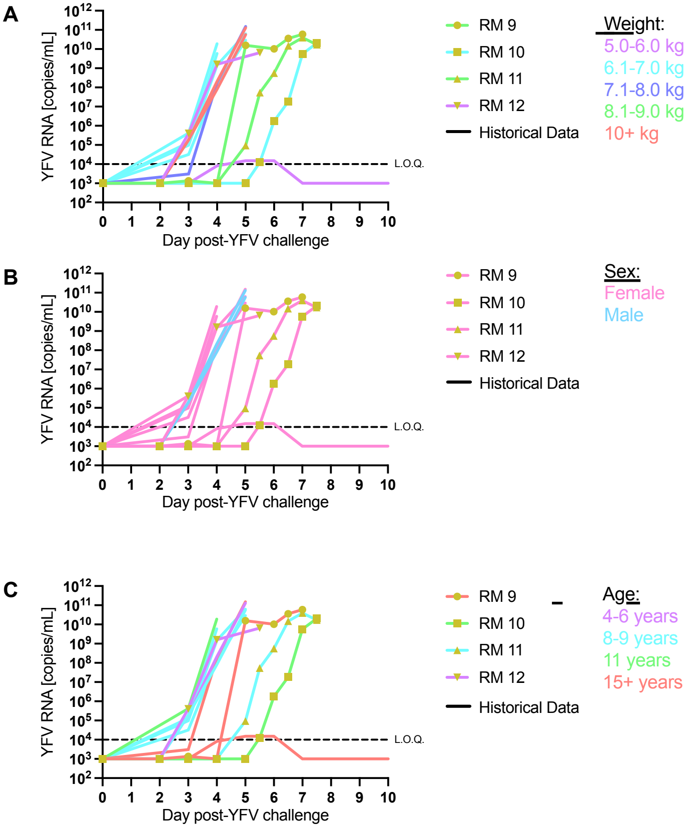

Supplemental Figure 1. Correlation between animal A) weight, B) sex, and C) age versus YFV viremia.

Supplemental Figure 2.

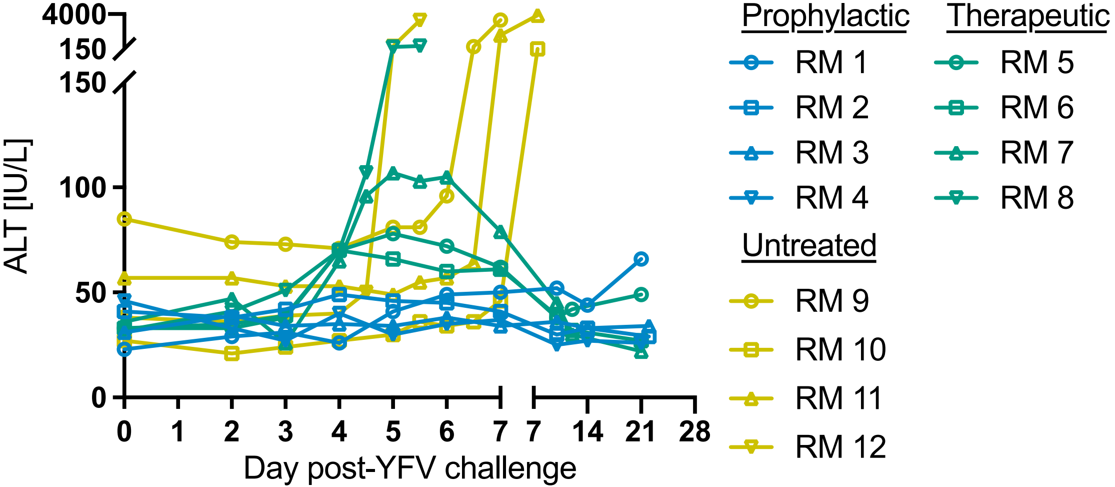

Supplemental Figure 2. ALT levels in RMs.

Supplemental Figure 3.

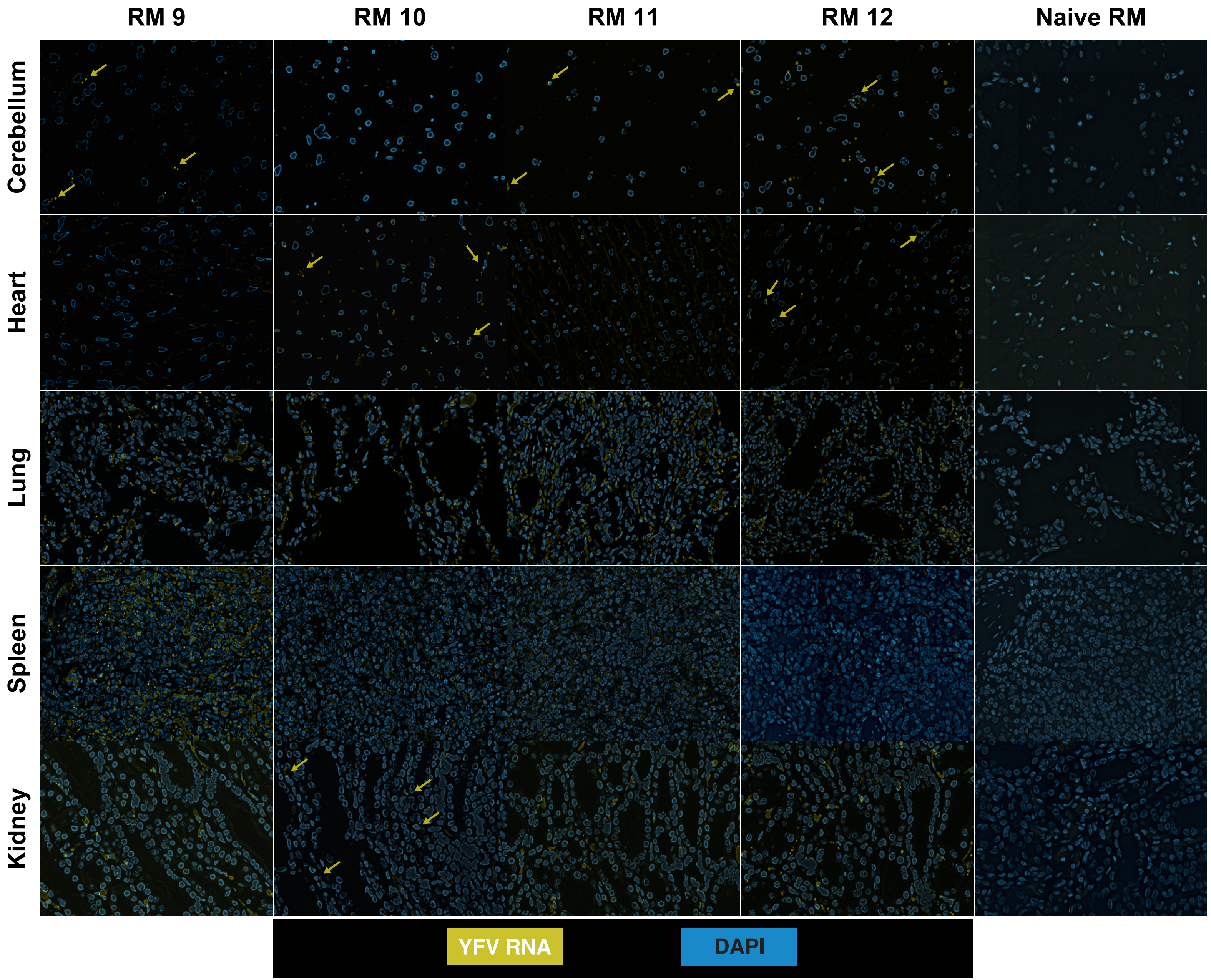

Supplemental Figure 3. RNAScope staining for YFV-DakH1279 in multiple necropsy tissues from untreated RMs.

Supplemental Figure 4.

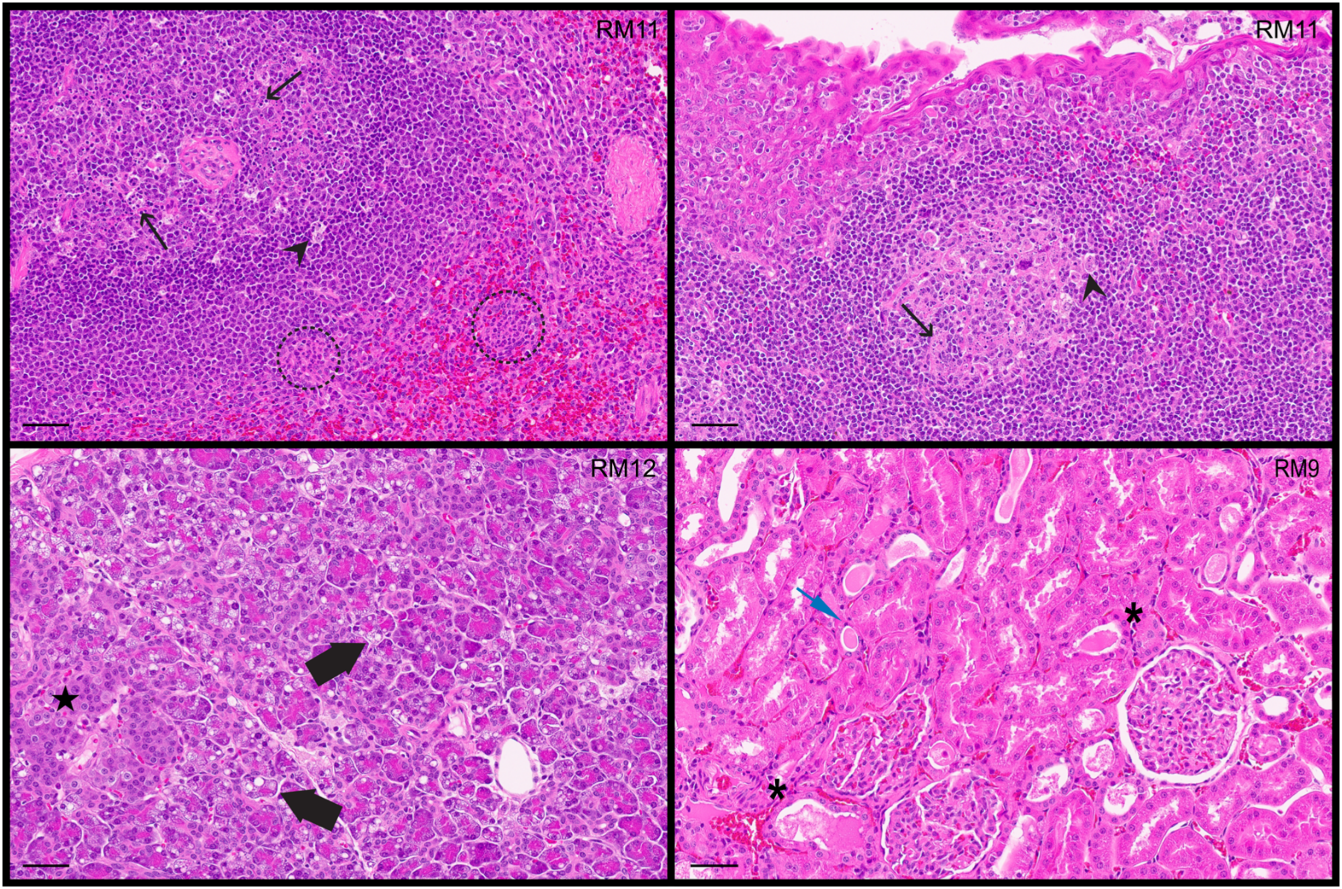

Supplemental Figure 4. Histolopathologic lesions in the spleen, tonsil, pancreas, and kidneys of YFV-DakH1279 infected RMs. Representative hematoxylin and eosin staining of the spleen (RM 11), tonsil (RM 11), pancreas (RM 12), and kidney (RM 9) of untreated RMs. Lymphoid necrosis (thin black arrow) and increased tingible body macrophages (arrowhead) in the germinal centers of the spleen and tonsils. Neutrophilic inflammation in the marginal zone and red pulp of the spleen (dashed circles). Vacuolation of acinar cells (thick black arrows) in the pancreas (Islet of Langerhans, star). Tubular epithelial cell degeneration and necrosis (blue arrow), ectasia, and proteinaceous casts (asterisks) in the kidneys. Scale bars, 100 µm.

Figure S5.

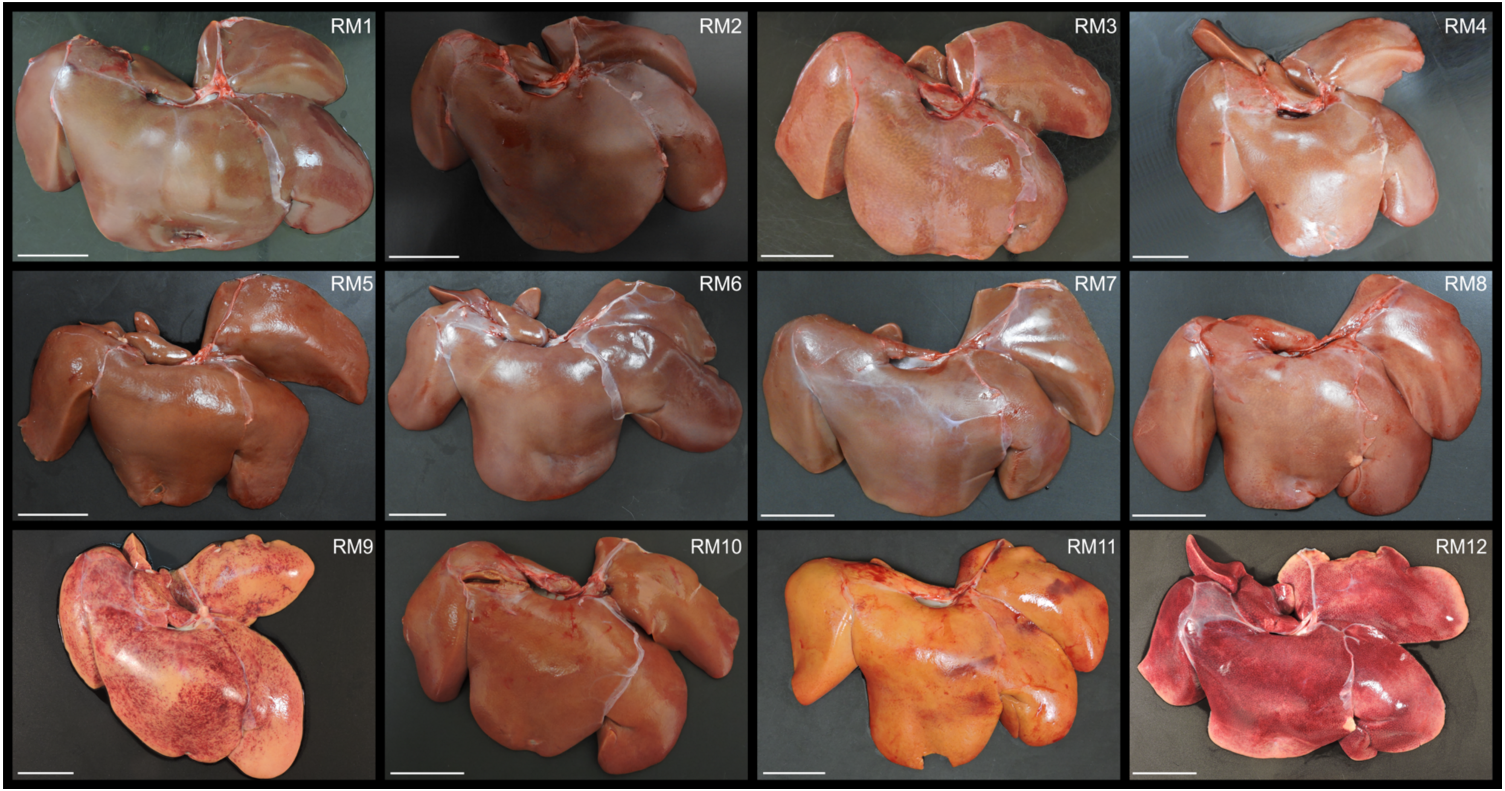

Figure S5. Gross findings in YFV-DakH1279-infected RMs. Gross images of livers from animals receiving prophylactic (RM 1-4), therapeutic (RM 5-8), or no treatment (RM 9-12). Untreated livers are variably pale yellow with hemorrhages. Scale bars, 3 cm.

Supplemental Figure 6.

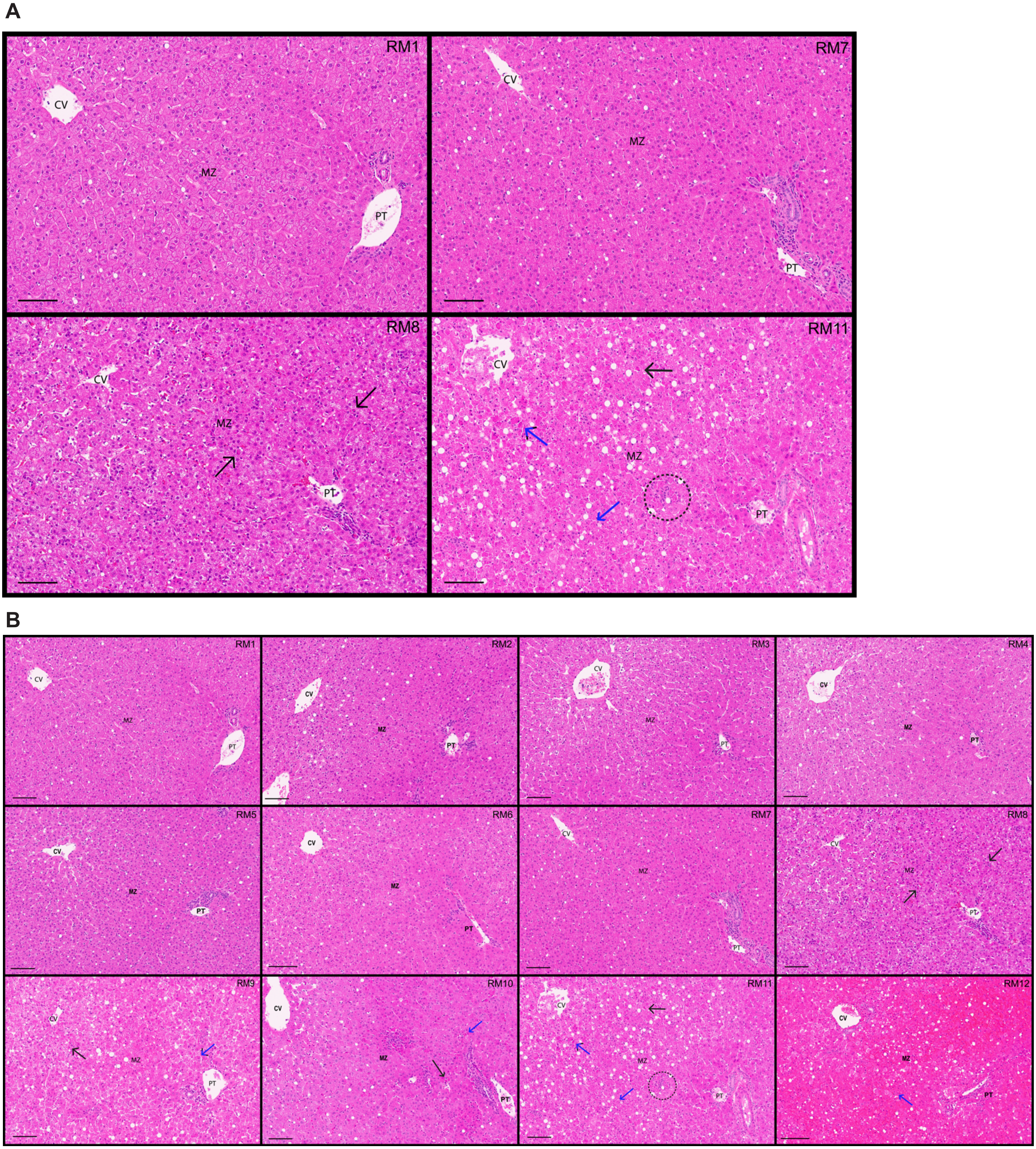

Supplemental Figure 6. Histological findings in YFV-DakH1279-infected RMs. A) Representative hematoxylin and eosin staining of livers in prophylactically treated (RM 1), therapeutically treated (RM 7, RM 8), and untreated (RM 11) RMs. Few midzonal Councilman bodies (black arrows) are in RM 8. RM 11 displays massive hepatic necrosis with degeneration (blue arrows), Councilman bodies, steatosis, and minor lymphocytic inflammation (dashed circle) in an untreated animal. Scale bars, 100 µm. PT, portal triad; MZ, midzonal region; CV, central vein. B) Hematoxylin and eosin staining of livers in prophylactically treated (RM 1-4), therapeutically treated (RM 5-8), and untreated (RM 9-12) RMs. Minimal midzonal degeneration with Councilman bodies (blue arrows) in RM 8 and a spectrum hepatic necrosis and degeneration (blue arrows), Councilman bodies, steatosis, and minor lymphocytic inflammation (dashed circle) in RM 9-12. Scale bars, 100 µm. PT, portal triad; MZ, midzonal region; CV, central vein.

ID	Experimental Group	Sex	Geographic Origin	Age (years)	Weight (kg)	Mamu-A1*001	Mamu-A1*002	Mamu -B*001	Mamu -B*008	Mamu -B*017
RM 1	Prophylactic	Female	India	12	11.35	NEG	POS	NEG	NEG	NEG
RM 2	Prophylactic	Female	India	12	10.9	NEG	NEG	ND	POS	POS
RM 3	Prophylactic	Female	India	12	10.1	NEG	NEG	ND	NEG	POS
RM 4	Prophylactic	Female	India	12	8.	NEG	NEG	ND	NEG	POS
RM 5	Therapeutic	Male	India	6	9.1	NEG	POS	NEG	NEG	NEG
RM 6	Therapeutic	Male	India	4	9.8	NEG	NEG	POS	POS	NEG
RM 7	Therapeutic	Male	India	4	7.4	POS	NEG	NEG	NEG	NEG
RM 8	Therapeutic	Male	India	3	5.4	NEG	NEG	ND	POS	NEG
RM 9	Untreated	Female	India	17	8.86	NEG	NEG	NEG	NEG	NEG
RM 10	Untreated	Female	India	11	6.6	ND	ND	ND	ND	ND
RM 11	Untreated	Female	India	9	8.15	NEG	POS	ND	NEG	NEG
RM 12	Untreated	Female	India	6	5.05	ND	ND	ND	ND	ND

Table S1. Demographics of study RMs. ND, No data.

Parameter	Degree of Parameter	Score
Overall clinical appearance, signs of hemorrhage, liver failure	Normal appearance, no petechial or ecchymotic hemorrhages	0
	Facial edema, photophobia, facial pallor	2
	Mild to moderate diarrhea or vomiting, mild dehydration, limited petechiae/ecchymosis, jaundice	5
	Persistent epistaxis, melena, retrobulbar hemorrhage, hematemesis, hypovolemia, shock	10
Respiratory rate, mucous membrane (MM) color, dyspnea	Normal signs (Respiratory rate = 32 to 50 BPM; MM pink)	0
	Mild (Respiratory rate = 51 to 65 BPM, with slightly increased effort; MM pale pink)	2
	Moderate (Respiratory rate = 66 to 80 BPM, with obvious difficulty breathing; MM muddy pink)	7
	Severe (Respiratory rate ≥ 80 bpm, respirations labored; MM blue)	10
Activity, attitude, appetite	Normal - Bright, alert, upright	0
	Mild depression, reduced appetite, head down, reduced interest in personnel and/or surroundings	3
	Recumbent when entering the room but gets up readily. Moderately reduced appetite.	5
	Recumbent when entering the room but gets up with stimulation at front of cage. Very disinterested in personnel. Hunched. Anorexic.	8
	Recumbent, will not get up even with excessive stimulation	10
Core temperature of anesthetized animal	>36.7°C (>98°F)	0
	36.7 – 35.4°C (98 – 95.6°F)	3
	35.2 – 34.1 (95.5 – 93.4°F)	6
	<34°C (<93.3°F)	10

Table S2. Clinical scoring rubric for study RMs.

RM ID	Liver	Spleen Apop/TM	Spleen Inflam	Lymphoid tissues	Kidneys	Pancreas	Gall bladder
RM 1	None	None	None	None	None	None	None
RM 2	None	None	None	None	None	None	None
RM 3	None	None	None	None	None	None	None
RM 4	None	None	None	None	None	None	None
RM 5	None	None	None	None	None	ND	None
RM 6	None	None	None	None	None	ND	None
RM 7	None	None	None	None	None	ND	None
RM 8	+	None	None	None	None	ND	None
RM 9	++++, H, L	+++	+++	+++	+++	+	+
RM 10	+++	None	None	None	None	None	None
RM 11	++++	++	+++	+	+	None	None
RM 12	++++, H	++	+	+	++	++	++

Table S3. Summary of histologic findings in the liver, spleen, lymphoid tissues, kidney, pancreas, and gall bladder. Liver +=severity of necrosis, H=hemorrhage, L=lymphocytic infiltration; Spleen apoptosis and tingible body macrophages +=severity of germinal center apoptosis/necrosis and tingible body macrophages, inflammation +=severity of neutrophilic inflammation; Lymphoid tissues +=severity of germinal center apoptosis/necrosis and tingible body macrophages; Kidneys +=severity of tubular epithelial degeneration and proteinaceous and/or cellular casts; Pancreas +=severity of acinar degeneration and loss of zymogen granules; Gall bladder +=severity of edema. Apop, apoptosis; TM, tingible body macrophages; Inflam, inflammation; ND, no data.

dpi	RM 9	RM 10	RM 11	RM 12
0.0	0	0	0	0
2.0	0	0	0	0
3.0	0	0	0	0
4.0	0	0	0	0
4.5	ND	ND	ND	0
5.0	0	0	0	0
5.5	0	0	0	7
6.0	0	0	0	
6.5	ND	0	0	
6.75	0	0	0	
7.0	12	0	10	
7.5		0	10	

Table S4. Clinical scores for control group animals. Observed total clinical scores for untreated RMs from the time of YFV infection until endpoint. dpi, days post-infection; ND, not done.
